# Aqueous Cold Sintering of Li-Based Compounds

**DOI:** 10.1021/acsami.3c00392

**Published:** 2023-04-13

**Authors:** Linhao Li, Jessica Andrews, Ria Mitchell, Daniel Button, Derek C. Sinclair, Ian M. Reaney

**Affiliations:** † College of Mathematics and Physics, Beijing University of Chemical Technology, Beijing 100029, China; ‡ Department of Materials Science and Engineering, University of Sheffield, Mappin Street, Sheffield S1 3JD, U.K.

**Keywords:** LLZO, LCO, cold sintering, solid electrolyte, ceramic composite, prefer orientation, textured ceramic

## Abstract

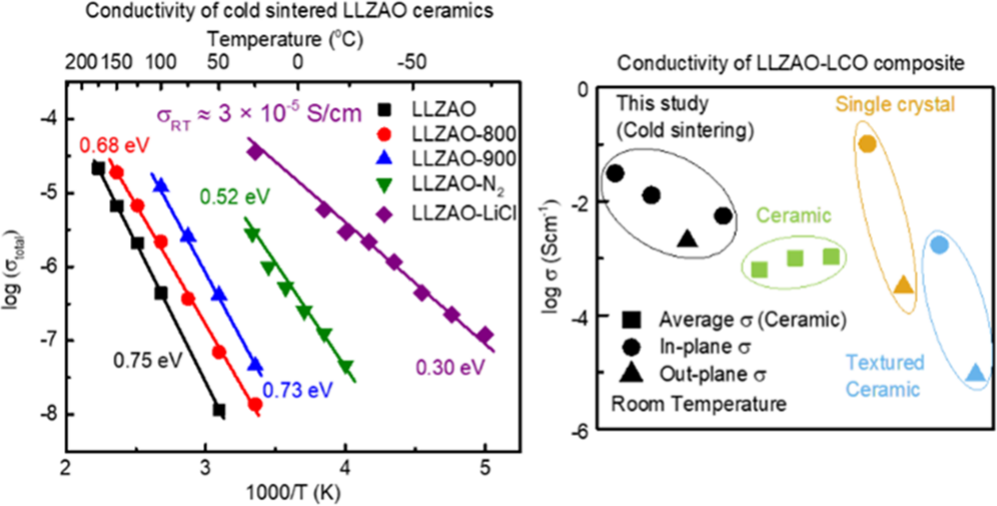

Aqueous cold sintering of two lithium-based compounds, the electrolyte Li_6.25_La_3_Zr_2_Al_0.25_O_12_ (LLZAO) and cathode material LiCoO_2_ (LCO), is reported. For LLZAO, a relative density of ∼87% was achieved, whereas LCO was sintered to ∼95% with 20 wt % LLZAO as a flux/binder. As-cold sintered LLZAO exhibited a low total conductivity (10^–8^ S/cm) attributed to an insulating grain boundary blocking layer of Li_2_CO_3_. The blocking layer was reduced with a post-annealing process or, more effectively, by replacing deionized water with 5 M LiCl during cold sintering to achieve a total conductivity of ∼3 × 10^–5^ S/cm (similar to the bulk conductivity). For LCO-LLZAO composites, scanning electron microscopy and X-ray computer tomography indicated a continuous LCO matrix with the LLZAO phase evenly distributed but isolated throughout the ceramics. [001] texturing during cold sintering resulted in an order of magnitude difference in electronic conductivity between directions perpendicular and parallel to the *c*-axis at room temperature. The electronic conductivity (∼10^–2^ S/cm) of cold sintered LCO-LLZAO ceramics at room temperature was comparable to that of single crystals and higher than those synthesized via either conventional sintering or hot pressing.

## Introduction

Interest in Li-ion compounds has surged over the last few decades, mainly driven by the development of various types of Lithium-Ion Batteries (LIBs). The use of solid-state electrolytes (SSEs) in LIBs is considered to improve battery safety and potentially achieve high power and energy density over a wide voltage and temperature window.^1,2^ However, due to the active chemical nature and volatility of lithium, the processing of Li compounds, especially oxides which require high temperatures for densification, is challenging.

Cold sintering is a newly developed low-temperature consolidation method for ceramics.^3,4^ It is pressure-assisted in the presence of a transient liquid phase that enables a dissolution–precipitation sintering mechanism.^5^ Due to its low temperature (<300 °C), cold sintering significantly reduces the energy consumed in densification compared with other routes.^6^ It also allows integration to high-density dissimilar materials such as ceramics and polymers. Since the first comprehensive publications on this technique in 2016 by Guo et al., it has drawn significant attention across various disciplines such as microwave ceramics, semiconducting oxides, ferroelectrics, ionic conductors, and Li battery materials.^7−21^

The garnet-structured Li_7_La_3_Zr_2_O_12_ (LLZO) is widely regarded as one of the most promising candidates as SSE,^22,23^ with its cubic polymorph (∼10^–3^ S/cm) more than two orders of magnitude more conductive than tetragonal polymorph (∼10^–5^ S/cm) in optimized ceramics.^24−26^ Consequently, most research has focused on stabilization of the cubic over the tetragonal polymorph, which may be achieved by using dopants on either the Li- (Al^3+^, Y^3+^, Ga^3+^, Fe^3+^) or the Zr-site (Nb^+5^, Ta^+5^, Sb^+5^).^27−36^ Unfortunately, conventional sintering requires 1000–1200 °C, often over long periods of time (up to 36 h in some studies), which subsequently leads to significant Li loss, secondary phases, and reduced density.^33,35,37^ Li loss may be compensated by adding extra Li_2_CO_3_ to the starting composition and/or using sacrificial powder(s), but the precise Li concentration remains an unknown variable (often graded from interior to exterior of the pellet) that is influential in controlling both the structure and ionic conductivity of LLZO.^38−41^ As a result, the highest values of conductivity for LLZO are only typically achieved in hot-pressed samples, which avoid Li loss by densifying within a closed system.

LiCoO_2_ (LCO) is a cathode material reported initially by Goodenough et al.^42,43^ Its high conductivity and stable structure still offer high energy density in comparison with newly proposed cathode materials such as LiMn_2_O_4_, LiFePO_4_, LiNi_1–*x*–*y*_Co*_x_*Mn*_y_*O_2_ (0 < *x*, *y* < 1), LiNi_0.80_Co_0.15_Al_0.05_O_2_, and *x*Li_2_MnO_3_·(1 – *x*)LiMO_2_ (M = Ni, Co, Mn, etc.).^44−52^ LCO possesses a layered rock salt structure (α-NaFeO_2_ type), which gives rise to its plate-like morphology and anisotropic conductivity that can differ by a factor of ∼500 between in-plane (a, b plane) and out-plane (*c*-axis) orientations based on single-crystal studies.^53^ As conductivity is critical for cathode materials, it is desirable to obtain textured LCO, which, until now, has been achieved either by processing a slurry in a strong magnetic field (e.g., 12 T)^54,55^ or via hot pressing.^56^ The former utilizes the magnetic anisotropy of LCO, whereas the latter is achieved through the application of high pressure and its action on the plate-like morphology.

There are generally two different approaches to the cold sintering of LLZO involving either aqueous^57^ or organic (e.g., dimethylformamide, DMF) transient solvents.^21^ Although relatively high density (80–90%) LLZO ceramics can be achieved, these two approaches have their own disadvantage. Aqueous cold sintering has been reported to give conductivity 3–4 orders of magnitude lower than optimized hot-pressed LLZO,^54^ whereas DMF is toxic, which adds process complexity for scale-up. In contrast, there are no reports of cold sintering of LCO despite its extensive use within the Li-ion battery technology.

In this study, Li_6.25_La_3_Zr_2_Al_0.25_O_12_ (LLZAO) was cold sintered to ∼87% relative density to give an optimized total conductivity of ∼3 × 10^–5^ S/cm, close to that of the bulk using a LiCl solution rather than pure water as the transient solvent. LLZAO was then used as a flux/binder to aid in the cold sintering of LCO. ∼95% dense 0.8LCO–0.2LLZAO cold sintered composites were obtained with a high degree of texture (Lotgering factor, LF = 0.9) at the pellet surface that yielded in-plane electronic conductivity comparable to single crystals at room temperature (10^–2^ S/cm).

## Experimental Procedure

LLZAO and LCO-LLZAO ceramics were prepared via cold sintering using raw powder supplied by Toshima (LLZAO, 99.9%) and Targray (LCO, 99.9%), respectively. For LLZAO, appropriate amounts of raw powder were weighed and mixed with 5 wt % of deionized water or 5 M LiCl solution using mortar and pestle for 10 min. The mixture was then placed in a 12.75 mm diameter stainless steel die, uniaxially pressed at 385–770 MPa, initially for 10 min at room temperature, and then for 30 min between 150–300 °C (heating rate of 10 °C min^–1^), and cooled to room temperature. For LCO-LLZAO, various compositions based on weight fractions of 1 – *x*(LiCoO_2_)–*x*(Li_6.25_La_3_Zr_2_Al_0.25_O_12_), (*x* = 0.2, 0.3, and 0.5) were prepared. Appropriate amounts of raw materials were weighed and mixed in acetone with a mortar and pestle for 20 min until completely dry to form a well-mixed powder. 5 wt % deionized water was then added and mixed with a mortar and pestle for a further 5 min. The mixture was placed in a 12.75 mm diameter die and cold sintered with the same parameters as LLZAO ceramics. In order to remove eventual traces of water, all pressed pellets were stored in an 80 °C oven for several days before tests. Pellet density was measured through a geometric method.

X-ray diffraction (XRD) was performed on a Bruker D2 phaser. Raman spectra were recorded using an InVia confocal Raman microscope on cold sintered pellets with a 20 kW power Green laser (514.5 nm radiation) for a 10 s exposure time. Microstructures of polished sections, parallel and perpendicular to the pressing direction, were recorded using either a Philips XL 30S FEG or FEI Inspect F scanning electron microscope (SEM). To prevent charging, the polished pellet surfaces were sputtered with Au using an Agar AGB7340 manual sputter coater.

Impedance spectroscopy, IS, was performed using an Agilent E4980A impedance analyzer with an Oxford cryo system on cold sintered ceramics pellets with sputter-coated Au electrodes. The IS measurements were carried out at an applied voltage of 100 mV, over the frequency range of 20 Hz to 1 MHz. Impedance data were corrected for sample geometry (thickness/area of the sample). High-frequency instrumental-related (impedance analyzer, leads, and sample jig) inductance effects were corrected by performing a short circuit measurement. High-temperature electrical conductivity was measured in air with a 4-probe contact method using a Netzsch SBA 458 Nemesis.

X-ray Computed Tomography (X-ray CT) scans were collected using a Zeiss Xradia 620 Versa X-ray Microscope (XRM). This method was employed specifically for nondestructive three-dimensional (3D) imaging. The sample was mounted (screwed) onto a Zeiss XRM sample holder without the need for any further sample preparation. X-rays were generated from a tungsten transmission target and collected on a CCD (charge-coupled device) 16bit 2000 × 2000 pixel detector camera. Scans were collected at an X-ray tube voltage of 150 kV, a tube current of 153 μA, 23 W, and an exposure time of 7 s per projection. 2401 projections were obtained over a scan time of ∼6 h. A filter (HE2) was used to remove low-energy X-rays, an objective lens gave an optical magnification of 40×, and binning was set to 4, producing an isotropic voxel (3D pixel) size of 740 nm at a field of view of 372 μm × 372 μm. A filtered back projection method reconstructed the data, and .txm files were converted to 8-bit grayscale two-dimensional (2D) .tiff stacks using Zeiss Scout and Scan Reconstructor software. The .tiff stack is made up of a series of images of varying grayscales, where low-density materials appear darker (black), and higher-density materials appear lighter (white). The collected data were then rendered with Dragonfly software by segmentation of LCO and LLZAO materials and reconstructed into a Mesh model.

## Results

### LLZAO

#### Cold Sintering LLZAO with Deionized Water

**Figure 1:**
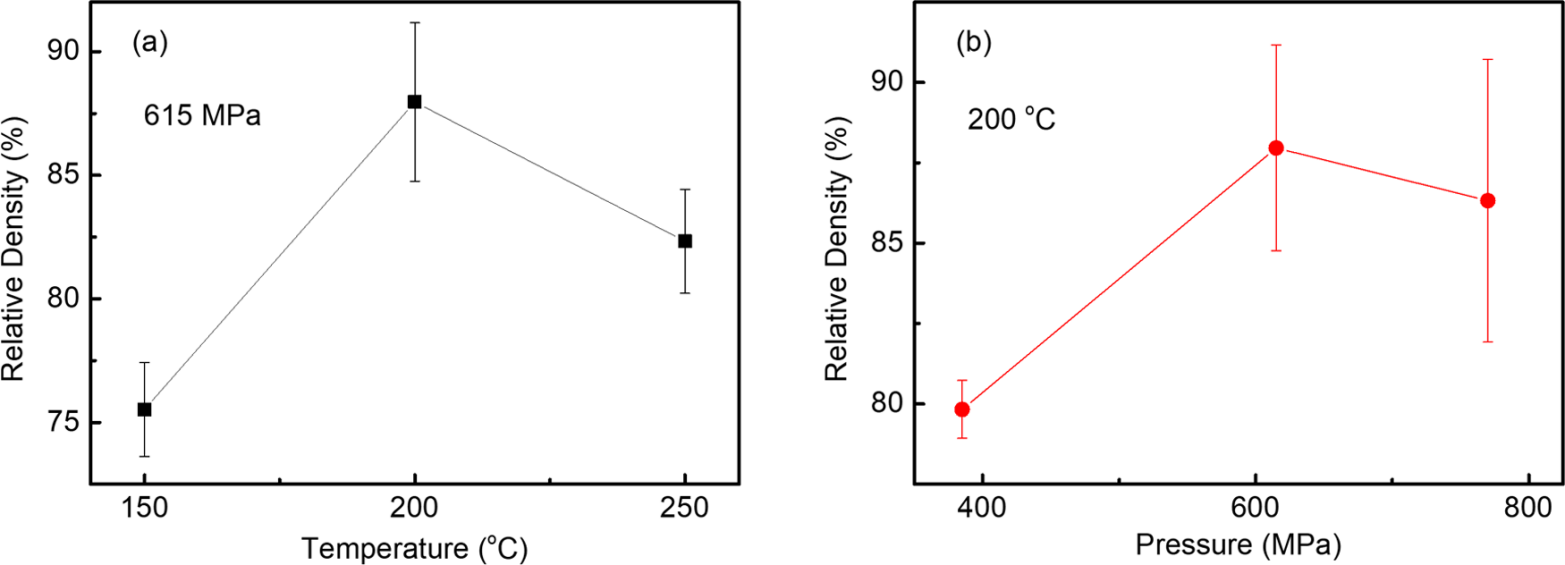
Relative density of the LLZAO ceramics sintered using various (a) pressures and (b) temperatures with deionized water.

The evolution of the relative density for LLZAO pellets with variable temperature and pressure using deionized water as a transient solvent is shown in Figure 1. The density varied from 3.86 to 4.57 g/cm^3^, which is 75–87% of the theoretical value (5.10 g/cm^3^), and attained a maximum at 200 °C, Figure 1a. Typically, LLZAO required a high pressure (>400 MPa), but no discernible difference was observed for pellets pressed at ∼600 and 750 MPa at 200 °C, Figure 1b. Optimum relative density (87%) was achieved at 615 MPa and 200 °C, and these conditions were used for further studies.

**Figure 2:**
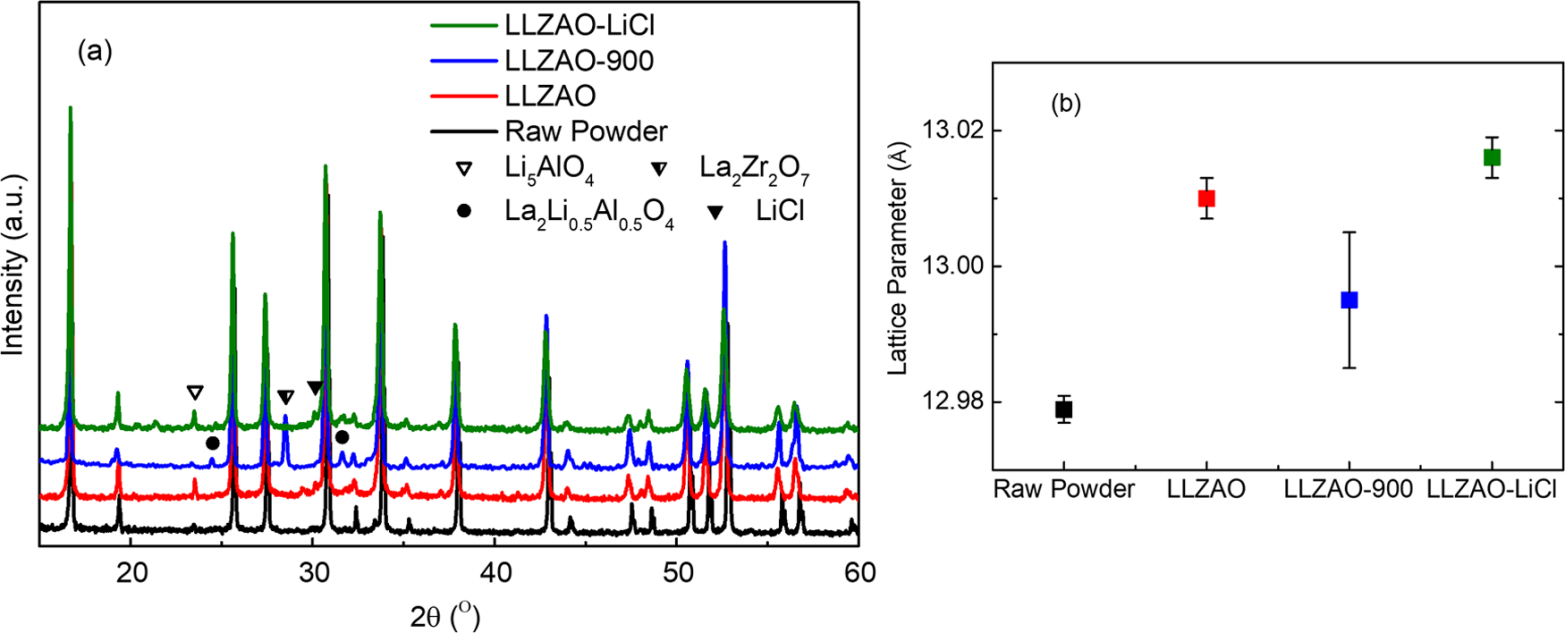
(a) Room temperature XRD patterns and (b) lattice parameters for raw powder, as cold sintered, 900 °C annealed, and cold sintered with LiCl LLZAO ceramics.

**Figure 3:**
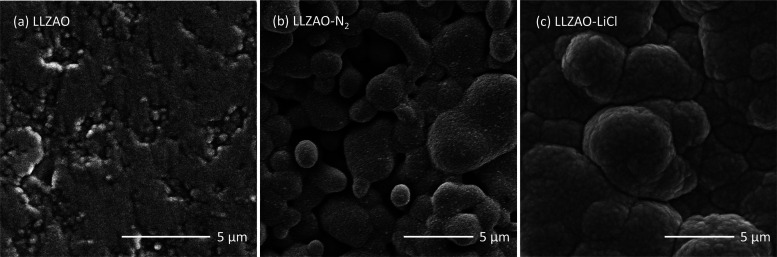
SEM backscattered images of variable LLZAO ceramics. (a) Cold sintered, (b) 900 °C annealed, and (c) as cold sintered with LiCl solution.

XRD patterns for raw powder and cold sintered LLZAO ceramics are shown in Figure 2a. Small peaks at ∼23.4 and 28.3° are associated with β-Li_5_AlO_4_ and La_2_Zr_2_O_7_, respectively. Peaks at ∼24.5 and 31.6° are associated with the La_2_Li_0.5_Al_0.5_O_4_ phase. The latter two phases are associated with Li loss and are present in samples heat-treated at 900 °C, in which volatilization is expected. In contrast, peaks associated with β-Li_5_AlO_4_ are strongest in samples in the as-cold sintered state, which has most likely formed at the particle–particle boundaries during densification using both deionized water and LiCl solution as the transient solvent. β-Li_5_AlO_4_ is reported to have a low decomposition reaction energy^58^ and using density functional theory (DFT) first-principles calculations, it is found to have a Li-ion conductivity on the order of 10^–4^ S/cm.^59^ The lattice parameter of the cubic LLZAO phase increased slightly from ∼12.97 Å for the raw powder to ∼13.01 Å after cold sintering, Figure 2b. Backscattered scanning electron images do not reveal any noticeable secondary phases, Figure 3a. A dense microstructure can also be observed with an average grain size in the range of 0.5–1 μm.

**Figure 4:**
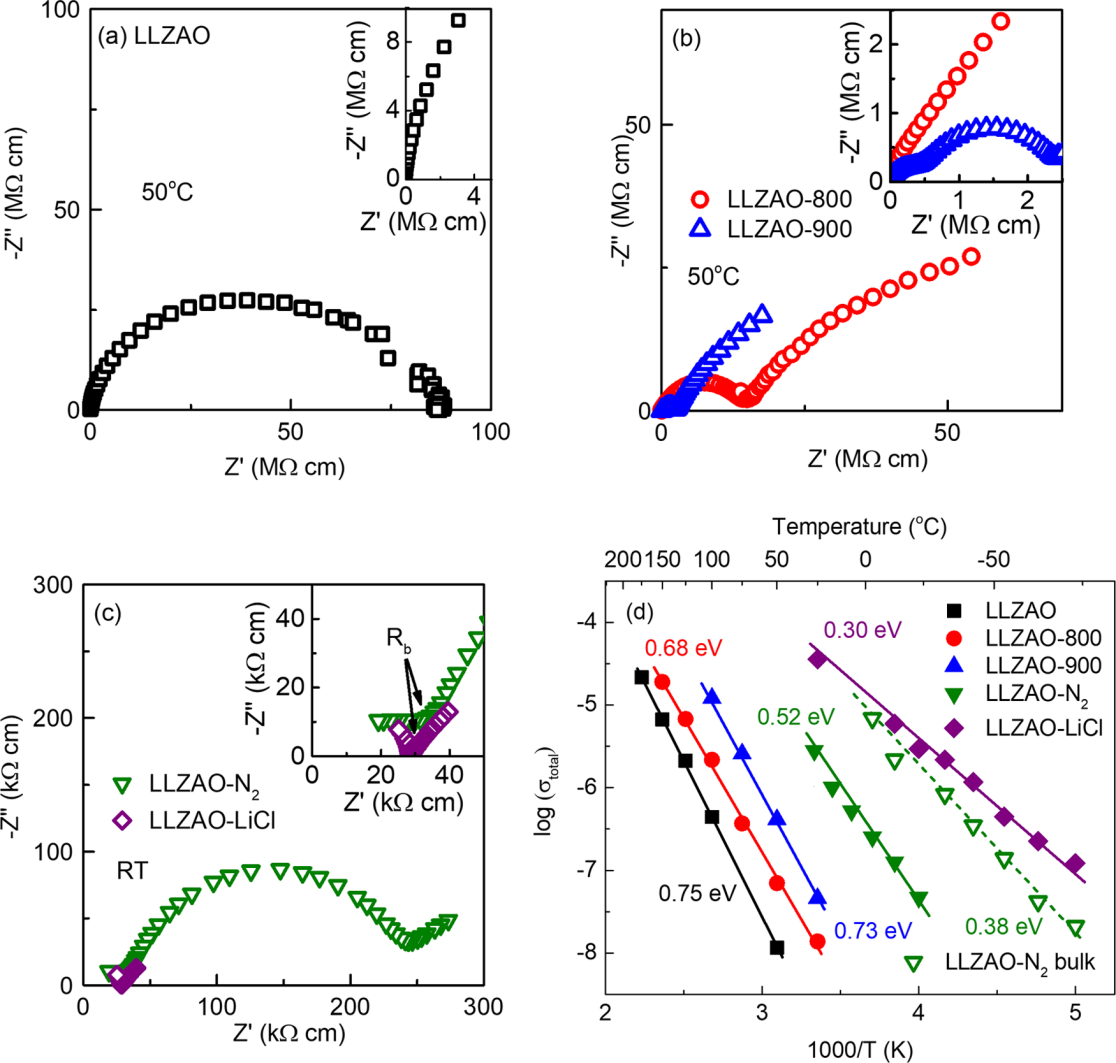
Z* plots of LLZAO ceramics (a) as cold sintered, (b) post-annealed in air at 50 °C, and (c) post-annealed in N_2_/cold sintered with LiCl solution at RT. (d) Arrhenius plot of total conductivity of LLZAO ceramics.

The conductivity and electrical microstructure of LLZAO ceramics were determined using impedance spectroscopy. A large asymmetric arc was observed in complex impedance (Z*) plots at 50 °C for LLZAO ceramics with a total resistivity, *R*_total_, of 8.6 × 10^7^ Ω·cm (total conductivity, σ_total_ = 1/*R*_total_ = 1.2 × 10^–8^ S/cm) taken from the low-frequency intercept of Z* plots, Figure 4a. As-cold sintered LLZAO, therefore, not only has a high *R*_total_ (4 orders of magnitude greater than optimized LLZAO) but also differs due to the absence of a low-frequency Warburg diffusion spike caused by Li-ion conduction.^28^ The Arrhenius plot of σ_total_ is shown in Figure 4d. The associated activation energy (*E*_a_) is 0.75 eV, which is also significantly higher than the reported values for solid-state sintered LLZAO of ∼0.4 eV.

It is unlikely that the bulk properties of LLZAO are affected by cold sintering; however, the electrical microstructure of LLZAO is dominated by a highly insulating grain boundary, which leads to much lower σ_total_, higher *E*_a_, and a different electrical microstructure to solid-state sintered counterparts. The main secondary phase peaks in XRD patterns in the as-cold sintered state arise from β-Li_5_AlO_4_ whose Li-ion conductivity is potentially as high as bulk LLZAO.^59^ It is therefore unlikely that β-Li_5_AlO_4_ is the blocking layer. Instead, we propose that Li_2_CO_3_ is present on the surface of LLZAO powder on exposure to ambient conditions, which is then retained on densification at the particle–particle interface or grain boundaries during cold sintering, acting as a blocking layer for Li-ion conduction.^60^

### Post-Annealing

To prove the hypothesis of the presence of blocking layers and improve the ionic conductivity, cold sintered LLZAO ceramics were annealed at 800/900 °C for 12 h in air (LLZAO-800 and LLZAO-900). No significant change in density was observed after annealing, as the temperature is significantly lower than that required for conventional sintering (1200 °C). The major peaks in the XRD patterns of LLZAO-900 (Figure 2a) could all be indexed according to LLZAO, but some low-intensity peaks of La_2_Zr_2_O_7_ and La_2_Li_0.5_Al_0.5_O_4_ were observed due to Li loss at high temperatures. The lattice parameter of LLZAO-900 decreased to 12.995 ± 0.010 Å, close to the value of the starting powder, Figure 2b, and SEM did not reveal evidence of secondary phase present in XRD patterns but did show an increase in the mean grain size (1–5 μm), Figure 3b.

Impedance results for the post-annealed samples are shown in Figure 4b. *R*_total_ at 50 °C decreases from ∼8.6 × 10^7^ Ω·cm (σ_total_ = 1.2 × 10^–8^ S/cm) for as-cold sintered LLZAO to ∼1.4 × 10^7^ Ω·cm (σ_total_ = 7.1 × 10^–8^ S/cm) for the LLZAO-800 and finally to ∼2.3 × 10^6^ Ω·cm (σ_total_ = 4.3 × 10^–7^ S/cm) for the LLZAO-900. However, the associated *E*_a_ remain relatively unchanged, 0.68 and 0.73 eV for LLZAO-800 and LLZAO-900, respectively. The electrical microstructure also evolved from one single arc for LLZAO to one arc and a low-frequency spike for LLZAO-800 and a second arc with lower resistivity resolved at a high-frequency range in LLZAO-900. Each of these arcs could be modeled, to a first approximation, on a single parallel resistor–capacitor (RC) element that were connected in series. For the LLZAO-900, the associated capacitances for the three components (two arcs and one spike) from high to low frequency are 1.7, 40 pF cm^–1^ and 52 nF cm^–1^, respectively. The three components can therefore be attributed to bulk, grain boundary, and Warburg diffusion, respectively. Samples were also post-annealed in N_2_ (LLZAO-N_2_) with the time extended to 24 hrs at 900 °C. Similar three electrical components were observed with *R*_total_ at RT further reduced to ∼2.4 × 10^5^ Ω·cm (σ_total_ = 4.2 × 10^–6^ S/cm) with *E*_a_ ∼ 0.52 eV, Figure 4c,d. It is noteworthy that the bulk resistivity, *R*_b_, of LLZAO-N_2_ at RT is 3.4 × 10^4^ Ω·cm (bulk conductivity, σ_b_, = 2.9 × 10^–5^ S/cm) with an associated *E*_a_ of 0.38 eV (dashed line in Figure 4d), similar to the reported values for solid-state sintered LLZO-based ceramics.

We conclude, therefore, that a highly resistive grain boundary component (blocking layer) dominates *R*_total_ and therefore σ_total_ of cold sintered LLZAO ceramics. These observations imply that the blocking layer decomposes on heating, consistent with the presence of Li_2_CO_3_ at the grain boundaries within samples cold sintered using deionized water as the transient solvent. The effect of the blocking layer can be reduced by post-annealing but not eliminated even in N_2_. An alternative approach is therefore proposed, in which a LiCl solution is used as the transient solvent (LLZAO-LiCl), following the same procedures described for deionized water.

### Cold Sintering with LiCl Solution

The LLZAO-LiCl ceramics were first cold sintered and then annealed at 900 °C for 12 h under N_2_. The relative density of LLZAO-LiCl ceramics is ∼80%, lower than with deionized water. As the change of theoretical density due to the presence of LiCl is less than 1%, the theoretical density of pure LLZAO is used here for the convenience of data processing. The XRD patterns of LLZAO-LiCl show extra peaks compared to LLZAO, attributed to LiCl, Li_5_AlO_4_, and La_2_Zr_2_O_7_ phase, Figure 2a. The calculated lattice parameter of the LLZAO phase is 13.016 ± 0.003 Å, Figure 2b, and SEM images suggest a similar particle size of 0.5–1 μm with no secondary phases observed, despite the extra peaks in XRD patterns, Figure 3c.

Z* plots of impedance data of LLZAO-LiCl consist of a single arc and a low-frequency spike, attributed to the bulk and Warburg diffusion, respectively. The *R*_total_ of LLZAO-LiCl at RT extracted from the complex impedance plane is 2.8 × 10^4^ Ω·cm (σ_total_ = 3.6 × 10^–5^ S/cm) with *E*_a_ of ∼0.3 eV, Figure 4c,d. Without the contribution from the blocking grain boundary, the overall ionic conduction of as-cold sintered LLZAO-LiCl is therefore improved dramatically compared to LLZAO-900 processed with deionized water, approaching values associated with solid-state sintered counterparts (∼10^–4/5^ S/cm) and similar to σ_b_ for LLZAO-N_2_ (2.9 × 10^–5^ S/cm).

### LCO-LLZAO Composites

**Figure 5:**
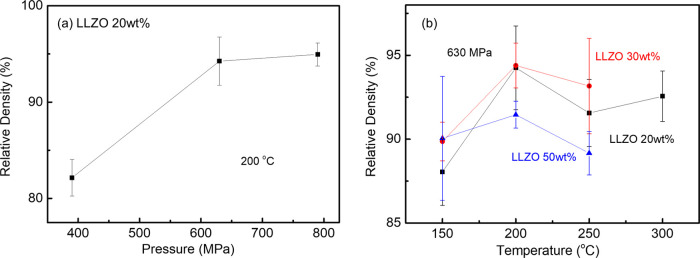
Relative density of LCO-LLZAO composites sintered with various (a) pressures and (b) temperatures for different amounts of LLZAO.

LLZAO powder was mixed into LCO powder as a binder/flux. The theoretical density of the LCO-LLZAO composites was calculated from the weight fraction of LCO and LLZAO according to
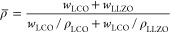
1where ρ̅, ρ_LCO_, and ρ_LLZAO_ are the theoretical densities of the composites, LCO and LLZAO and *w*_LCO_ and *w*_LLZAO_ are the weight fractions of LCO and LLZAO, respectively. Figure 5 shows the relative density of the LCO-LLZAO composite ceramics sintered with different LLZAO wt fractions, pressures, and sintering temperatures. Generally, the density of LCO-LLZAO composites increases initially with increasing pressure and temperature up to 200 °C and 630 MPa and then remains unchanged. Above 20 wt %, the wt fraction of LLZAO has little impact on the density, suggesting that the LLZAO acts as a binder/fluxing agent whose concentration should be minimized to maximize the electrical properties of the composite cathode material. 0.8LCO–0.2LLZAO cold sintered at 200 °C at 630 MPa was therefore chosen as the optimized composition for further tests and, for simplicity, will be referred to as LCO-LLZAO in the following sections.

**Figure 6:**
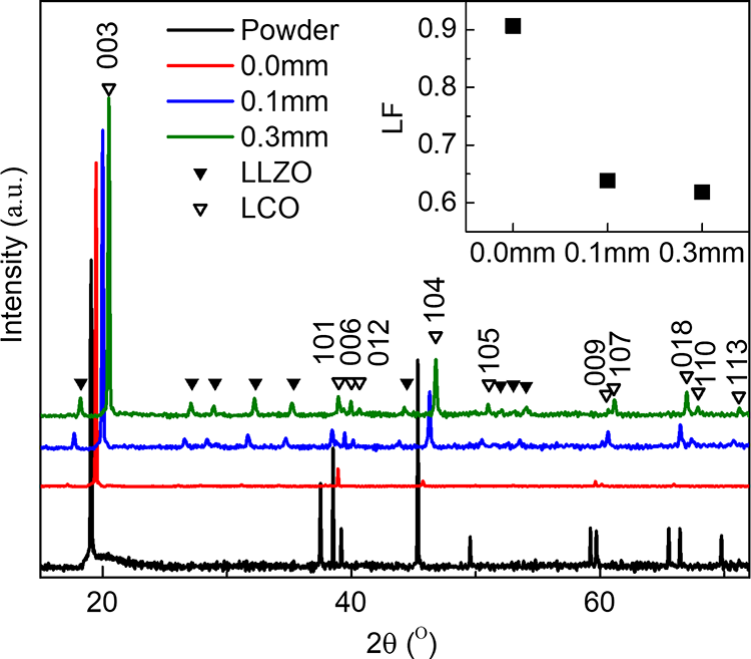
Room temperature XRD data of LCO powder and unpolished/polished cold sintered LCO-LLZAO ceramics. The length (*x*-axis) in the inset indicates the thickness of the layer removed and the associated Lotgering factor (LF).

XRD data on cold sintered ceramics revealed peaks attributed to LCO and LLZAO, Figure 6, but due to the relatively small wt %, it is not possible to establish whether the LLZAO is present as the tetragonal or cubic polymorph. The peak intensities for LCO are different from the randomly orientated powder, with significantly enhanced intensities for planes parallel with (001) peaks, indicating the ceramic has become textured. However, the peak intensities of polished (0.1 and 0.3 mm depth, respectively) and unpolished (0.0 mm depth) surfaces differ, illustrating the texture varies with depth away from the cold sintered surface.

The Lotgering factor (LF) indicates the degree of texture and is defined as
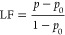
2where *p* is the fraction of the sum of peak intensities, which belongs to a particular orientation to that of the summation intensity of all peaks, and *p*_0_ is *p* of the associated peaks from a randomly oriented sample.^61^ LF = 0 and 1 correspond to random and complete orientation, respectively. The calculated LFs for LCO-LLZAO ceramics are shown in the inset of Figure 6. For an unpolished surface, LF = 0.91, but on polishing the sample surface, this decreases to ∼0.63 and 0.61 on removing 0.1 and 0.3 mm of material, respectively. The calculated lattice parameters for the LCO phase in cold sintered LCO-LLZAO are *a* = 2.8159 (7) Å and *c* = 14.049 (3) Å.

**Figure 7:**
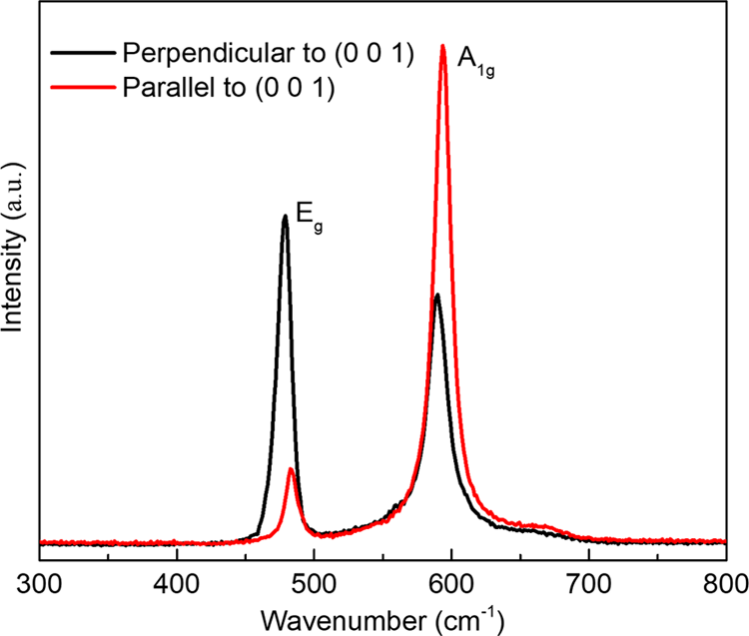
Raman spectra of LCO-LLZAO recorded from different orientations.

Raman spectra recorded parallel and perpendicular to the (001) direction are shown in Figure 7. Only two Raman active modes (E_g_ and A_1g_) were observed between 300 to 800 cm^–1^, both of which can be attributed to the LCO phase.^56^ The intensity ratios of *I*_E_g__/*I*_A_1g__ are 0.18 and 1.3 for the parallel and perpendicular directions, respectively, thus indicating the ratio increased with a lower degree of the *c*-axis orientation in the latter case. Furthermore, the absence of a clear peak at 690 cm^–1^ associated with Co_3_O_4_ suggests this possible impurity phase is not present in cold sintered LCO-LLZAO,^62^ consistent with XRD data.

**Figure 8:**
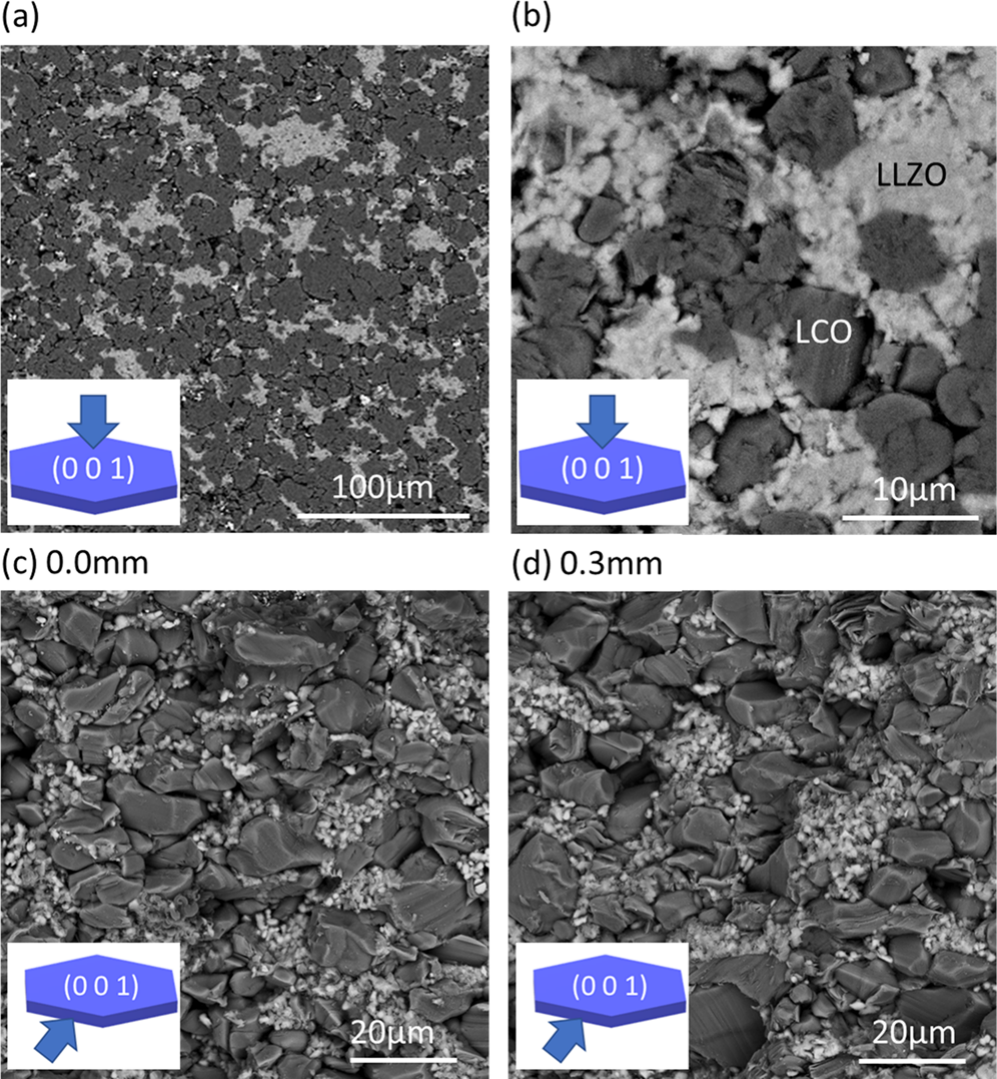
SEM backscattered images of LCO-LLZAO ceramic taken (a), (b) parallel and (c), (d) perpendicular to the (001) direction. Panels (c and d) are obtained from approximately 0.0 and 0.3 mm below the pellet surface, respectively.

Backscattered SEM images of the ceramics revealed a mixture of dark (lower weight average atomic number, WAA) LCO and bright (higher WAA) LLZAO phases. Images of the as-pressed pellet surface show that the (001) facets (highlighted) of the plate-like crystals of LCO dominate, Figure 8a,b, whereas for images obtained normal to the as-pressed surface, the (001) facets of the LCO crystals are largely absent, Figure 8c,d, consistent with XRD data. The average grain sizes are ∼5–20 and <1 μm for LCO and LLZAO, respectively.

**Figure 9:**
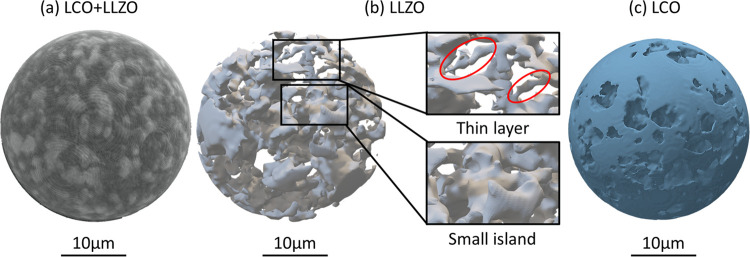
(a) 3D X-ray CT renderings of LCO-LLZAO composite ceramics. Mesh model of the distribution of (b) LLZAO and (c) LCO phases.

The SEM images in Figure 8 only provide information of a 2D cross section of the microstructure of LCO-LLZAO ceramics. As an electrolyte material, the electronic conductivity in LLZAO is negligible compared to its ionic conductivity. For the LCO-LLZAO composite to work as a cathode with high electrical conductivity, it is essential to ensure the LLZAO phase has not formed a continuous blocking layer(s). 3D imaging using X-ray CT was therefore employed to determine the distribution of both phases, Figure 9. Due to the relatively low resolution of X-ray CT (voxel ∼740 nm) compared to the SEM imaging adopted in this study (nm level), LCO and LLZAO grains cannot be clearly distinguished within their clusters. Nevertheless, the two different phases can easily be identified from the X-ray CT imaging, with the lower-density (darker) areas representing LCO and higher-density (lighter) areas representing LLZAO, Figure 9a. Mesh models of both phases have then been extracted and show clearly different morphologies between the two phases. For the LLZAO phase, two morphologies have been observed, i.e., thin layers and small islands, Figure 9b. The former corresponds to LLZAO phase along the LCO grain boundary (between 2 grains), and the latter corresponds to pockets between LCO grains (3 grains or more). On the other hand, the distribution of the LCO matrix seems continuous, with some small voids, confirming the availability of pathways for electrical current.

In this study, two different methods have been adopted to measure the conductivity of the LCO-LLZAO composite: IS and a 4-probe contact method. The former is useful for establishing ionic and/or electronic conductivity and to determine the electrical microstructure of electroceramics; however, the conductivity of LCO is too high for IS above ambient temperatures. Consequently, IS and the 4-probe contact method were used below and above room temperature, respectively.

**Figure 10:**
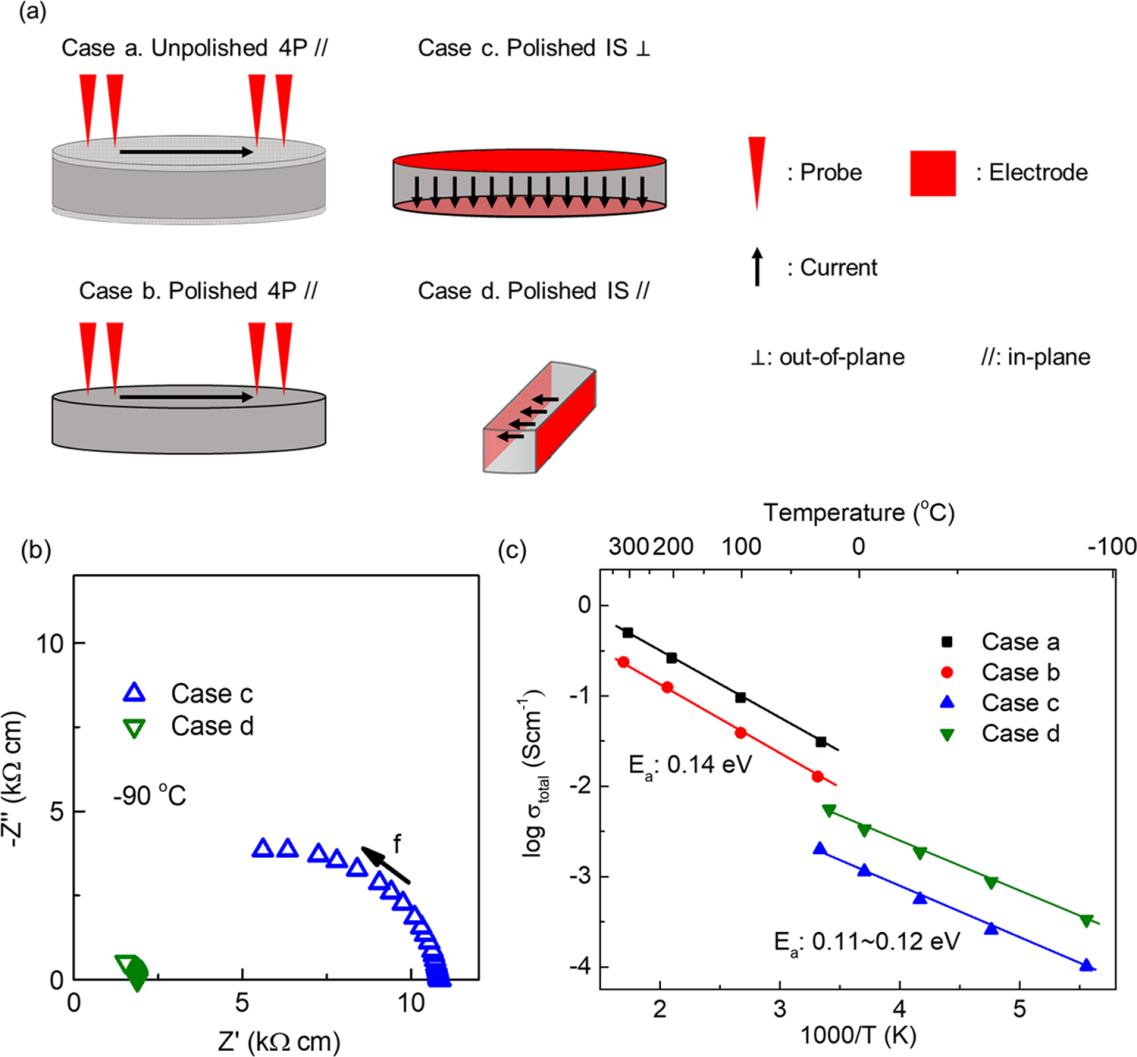
(a) Schematic illustrations of the experimental setups for the 4-probe method and impedance spectroscopy (polished: the top 0.1 mm layer polished off from both sides of the sample). (b) Z* plots of polished LCO-LLZAO ceramics measured along in- and out-of-plane directions. (c) Arrhenius-type plots of total conductivity for unpolished and polished LCO-LLZAO ceramics measured along in- and out-of-plane directions.

The electrical properties of LCO are anisotropic with “in-plane” (a, b plane) 500× greater than the “out-plane” (*c*-axis) conductivity.^53^ As described above, cold sintered LCO-LLZAO pellets possess a complex microstructure with highly textured surface grains (LF = 0.91), whereas those toward the center are less oriented (LF = 0.6). The conductivity of cold sintered LCO-LLZAO ceramics, therefore, depends on both the direction and region (pellet center vs surface) measured. To distinguish the conductivity in different directions and regions, polished (0.1 mm removed) and unpolished LCO samples were measured through thickness and along their diameter. The conductivity of pellets measured through thickness is dominantly out of plane, particularly near the surface, whereas through their diameter, conductivity through the a and b plane is favored. Schematics detailing the geometry of the electrical measurements are shown in Figure 10a.

Z* plots of polished LCO samples measured from both in- and out-plane directions are shown in Figure 10b, cases d and c, respectively. Only one incomplete arc is present in the measured temperature range (−90 to 25 °C) in both cases. The absence of a low-frequency response indicates dominant electronic conduction. The total resistivity, *R*_total_, of samples were taken from the lowest frequency intercept on the *Z*’ axis.

Total conductivity, where σ_total_ = 1/*R*_total_ was extracted from impedance measurements and is shown in Figure 10c, together with σ_total_ measured on samples using the 4-probe contact method at elevated temperatures. The conductivity measured from different directions/regions of the samples shows anisotropic behavior with a maximum difference (cases a and c) of more than one order of magnitude. Similar to work reported on single crystals, out-of-plane conductivity (case c) is lower than in-plane conductivity (cases a, b, and d), with the 4-probe method on unpolished samples exhibiting the highest conductivity. The activation energy for conduction, *E*_a_, is similar for all samples with a value of ∼0.11–0.14 eV, which is characteristic of electronic conduction in LCO and agrees with the IS data.

**Figure 11:**
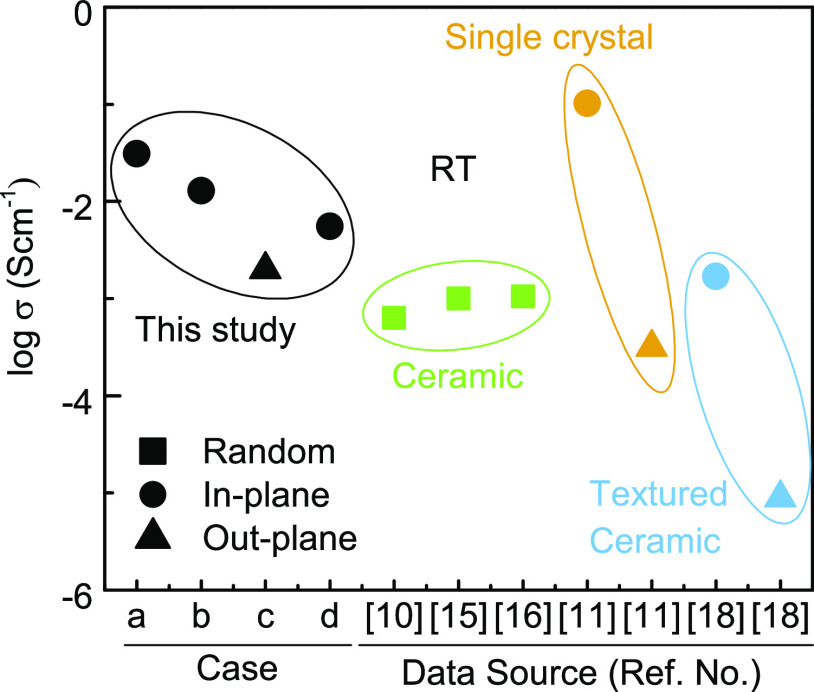
Comparison of room temperature conductivity of cold sintered LCO-LLZAO pellets in this study (case a: in-plane 4-probe surface layer; case b: in-plane 4-probe inner layer; case c: out-plane IS; case d: in-plane IS) with conventionally sintered LCO ceramics (random: refs (10, 15) and (16); textured: ref (18)) and single crystals (ref (11)).

A comparison of σ_total_ for LCO-LLZAO in this study and other studies is shown in Figure 11. All σ_total_ obtained in this study are high compared to previous studies, with the exception of the in-plane conductivity obtained from single crystals, which is greater (case a) by a factor of 3.^53,56,63−65^ We note, however, that even the out-of-plane conductivity in our study (case c) is higher than that reported in untextured LCO ceramics prepared by a high-temperature conventional densification route. Hot pressing is known to induce texture in LCO ceramics with LF = 0.96,^56^ but the conductivity remains anomalously low, suggesting other factors such as highly resistive grain boundaries may dominate the conduction properties.

## Discussion

### LLZAO

The dissolution–precipitation mechanism associated with cold sintering is easier to achieve in systems that exhibit congruent dissolution and high solubility.^9^ Xinchao et al. suggest that LLZAO undergoes only incongruent dissolution since Li^+^ possesses a much higher solubility than the other constituents, La^3+^, Al^3+^, and Zr^4+^.^57^ As a result, a β-Li_5_AlO_4_ phase was observed between grains (i.e., along grain boundaries), acting in part as a binding phase, which helps densification of the ceramic. In the case of LLZAO, β-Li_5_AlO_4_ has also been observed in XRD as a secondary phase. We therefore propose that the high density (87%) achieved by cold sintering of LLZAO with deionized water indicates a similar mechanism in this study. However, the use of a LiCl solution partially inhibits the dissolution of Li^+^ from the LLZAO particles and reduces the formation of the β-Li_5_AlO_4_. Simultaneously, LiCl precipitates at the particle boundary during evaporation, acting as a bond phase. The reduction in density for samples with LiCl solution rather than deionized water as the transient phase suggests that β-Li_5_AlO_4_ is a more effective bond phase than LiCl in the densification of LLZAO.

The starting powder and LLZAO-900 possess smaller lattice parameters than the as-cold sintered LLZAO and LLZAO-LiCl, Figure 2b. This effect is attributed to the H^+^/Li^+^ exchange mechanism. Larraz et al. studied the role of hydration in LLZAO and reported that the insertion of a proton causes a mild lattice expansion, which also exists in other related systems such as Li_7_La_3_Sn_2_O_12_.^26^ During cold sintering of LLZAO, the insertion of a proton leads to a slight increase in lattice parameter, which is reversed on heat treatment at 900 °C as the H^+^ is evolved and returns to a similar value as the starting powders.

σ_b_ in the LLZAO-N_2_ and LLZAO-LiCl ceramics have similar values, and it is concluded that the bulk conductivity of LLZAO ceramics is largely unaffected by cold sintering, Figure 4c. In contrast, σ_total_ varies dramatically, and the associated *E*_a_ of LLZAO, LLZAO-800, and LLZAO-900 (∼0.7 eV), which is dominated by an insulating grain boundary component are significantly higher than the reported values of ∼0.26–0.41 eV for LLZAO ceramics, Figure 4d.^25,28,33^ Due to the reduced grain boundary response, LLZAO-N_2_ has an intermediate σ_total_ and *E*_a_ (0.52 eV). The smaller σ_total_ in LLZAO, LLZAO-800, LLZAO-900, and LLZAO-N_2_ is therefore attributed to an insulating grain boundary component (Li_2_CO_3_) most likely inherent on the particle surfaces prior to densification.

LLZAO is known to rapidly form a layer of Li_2_CO_3_ on its surface during exposure to air, but it starts to decompose to Li_2_O and CO_2_ above 730 °C.^66^ Conventional sintering occurs significantly above this temperature, and any residual Li_2_CO_3_ formed on the particle surfaces during storage and pressing is removed through decomposition and subsequent evolution of CO_2_ with Li re-entering the LLZAO lattice. However, this mechanism cannot occur in cold sintered samples, and Li_2_CO_3_ is residual in the pellet interior for samples that use deionized water as the transient solvent. Annealing cold sintered samples at 900 °C, especially under N_2_, promotes decomposition of the Li_2_CO_3_ and decreases the grain boundary contribution to the total resistivity, increasing the total conductivity of the samples. However, a significant grain boundary contribution still exists even in LLZAO-N_2_, Figure 4c. This is mainly due to the density difference between a normal green ceramic and a cold sintered ceramic. As-pressed green bodies possess a relative density of ∼60–70%. At the sintering temperature, therefore, all particle surfaces are connected via microchannels to the grain exterior, allowing egress of CO_2_. However, cold sintered samples have a high relative density (∼87%) before annealing; hence, egress of CO_2_ from regions of closed porosity is problematic, and residual Li_2_CO_3_ remains at the grain boundaries.

The use of LiCl solutions rather than deionized water as a transient solvent appears to suppress the grain boundary blocking layer, virtually eliminating its contribution to σ_total_. The mechanism for suppression of the blocking layers via LiCl solutions remains to be elucidated but may relate to the formation of an intermediate dilithium carbonate hydrochloride (CHClLi_2_O_3_)^67^ compound during the unusual conditions during cold sintering. Nonetheless, the use of LiCl solutions is an effective means of cold sintering LLZAO ceramic electrolytes and could, in the future, result in a facile methodology for the fabrication of thick films suitable for the fabrication of oxide solid-state batteries.

### LCO-LLZAO

The conductivity of LCO-LLZAO ceramics in this study was measured via two techniques, impedance spectroscopy and a 4-probe contact method. Although they both measure *R*_total_, the sampling regions are different. As illustrated in Figure 10a, the current/electric field of IS measured samples (cases c and d) goes through the whole sample, whereas for the 4-probe method, data is obtained primarily from a thin surface layer (cases a and b). The 4-probe method on unpolished samples (case a) therefore gives the highest conductivity as data are obtained only from highly textured regions (LF = 0.91) and are thus comparable with single crystals. Cases b, c, and d measure the conductivity from samples with the highly textured surface layer removed. Case b has lower conductivity than case a due to the lowering of the LF value on removal of the surface layer. We attribute the small difference in conductivity between cases b and d (same orientation) to the modest LF (∼0.6), which develops in the interior of the pellet during cold sintering.

Comparison of LCO conductivity in this study to previous work indicates the LLZAO phase has not formed a blocking layer, which confirms the results from SEM and X-ray CT, Figures 8 and 9, respectively. Moreover, despite an LF ∼0.6 in the pellet center, the out-of-plane conductivity measured in case c is higher than for conventionally sintered LCO ceramics with LF ∼ 0. This result cannot be explained through texture, which predicts a lower conductivity, Figure 11, indicating the enhancement of conductivity must be due to another/other factor(s). One possible source could be the presence of highly conducting Co_3_O_4_ formed during the CSP; however, Raman spectroscopy showed no evidence of the presence of this phase, Figure 7. Two other plausible mechanisms are: (i) a space charge model and (ii) a change in the Li concentration in the LCO main phase.

#### Space Charge Model

Enhanced ionic conductivity has been widely observed in two-phase solid systems based on a space charge layer (SCL) at the interfaces between the different phases, i.e., an ionic conductor with an insulator or two ionic conductors. Liang et al. demonstrated that addition of Al_2_O_3_ to LiI increases the ionic conductivity by a factor of ∼50 at room temperature.^68^ Maier et al. subsequently discussed the mechanism of enhancement of ion transport in the SCL region,^69−71^ and Braun et al. derived a mathematical model for the SCL formation from first principles.^72^ Over several decades, there have been various reports of the SCL effect on a variety of ionically conducting materials, i.e., not limited to Li-based materials. In our study, the fine-scale distribution of the LLZAO phase with the LCO matrix, combined with the fact that LLZAO itself can exhibit high levels of Li-ion conduction, could generate SCLs in these composite ceramics.

#### Li-Ion Concentration Model

During cold sintering, a small fraction of Li-ions could leach into the water and may not re-enter the LCO lattice due to the low processing temperature of 200 °C. The LCO matrix may become Li-deficient with charge balance maintained by either creation of oxygen vacancies, interstitial cations (Li/Co), or the formation of electron holes. The first two mechanisms are improbable as both require relatively high energy of formation (temperature) to obtain and have negligible influence on the conductivity at room temperature. In contrast, the third mechanism is generated via a well-known low-energy process common in cobaltates in which there is an increase in transition-metal valence from 3+ to 4+, according to the following equations:
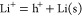

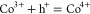
The oxidation of LCO should lead to enhanced p-type conduction due to an increase in the mixed oxidation state Co^3+^ and Co^4+^ ions. Ménétrier et al. proposed a relationship between Li concentration and the lattice parameter of LCO^73^ for which our study (*a* = 2.8159 (7) Å and *c* = 14.049 (3) Å) suggests a Li stoichiometry of ∼0.98–1.00 and therefore indicates only low Li loss (if any) during the CSP. Nonetheless, the greater sensitivity of electrical measurements to variations in p-type conductivity is consistent with the Li-ion concentration model.

These two mechanisms are not necessarily mutually exclusive, and improvement in both electronic and ionic conduction is possible. However, the absence of a low-frequency spike in the Z* plots, Figure 10b, is an indicator of Warburg diffusion, and therefore, ion migration at the sample/electrode interface indicates the conduction is predominately electronic. Furthermore, the low *E*_a_ associated with σ_total_ also supports a model based primarily on electronic conduction. Therefore, the space charge model is unlikely to be the sole mechanism responsible for enhanced conductivity.

It has been suggested the temperature-dependent in-plane conductivity of LCO is consistent with a variable range-hopping (VRH) mechanism instead of the normal activation (Arrhenius-type) mechanism. Takahashi et al.^53^ reported a two-dimensional (2D) VRH mechanism for single crystals, whereas Zhang et al.^56^ reported a three-dimensional (3D) VRH mechanism for highly textured ceramics. The latter attributed the 3D VRH mechanism to the role of grain boundaries and/or the nonperfect alignment of grains in their textured ceramics. According to VRH theory, the conduction should obey
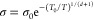
where *d* is the dimension of the conduction path and *T*_0_ is the Mott characteristic temperature. The conductivity data in this study were therefore plotted against 1000/*T*, *T*^–1/3^, and *T*^–1/4^ to verify which mechanism(s) is adopted in cold sintered samples, Figures S1 and S2. Based on the residual sum of squares (RSS) associated with linear fitting, the sub-ambient conductivity data of both in- and out-of-plane conductivity is consistent with a VRH mechanism. The out-of-plane conductivity (case c) shows a better fit for 3D VRH, whereas it is difficult to decipher between 2D and 3D VRH for the in-plane conductivity (case d) due to the lack of data points. The in-plane conductivity was remeasured to collect additional data, Figure S3, and fitting to 3D VRH generates a lower RSS than to 2D VRH. In contrast, the RSS of in-plane conductivity at elevated temperatures (cases a and b) has its lowest value when plotted against 1000/*T*, Figure S2. This indicates the in-plane conductivity of cold sintered LCO-LLZAO composites at higher temperatures is better described by the normal activation process rather than VRH.

## Conclusions

The present work suggests densification of LLZAO via an aqueous cold sintering approach is not only feasible but can also achieve conduction similar to that of the bulk (∼3 × 10^–5^ S/cm) or raw powder (i.e., no other resistive component introduced) if a LiCl solution is used instead of deionized water. If only deionized water is utilized, the relative densities (87%) of cold sintered samples are higher, but highly insulating grain boundary blocking layers form, which are not completely eliminated even after a post-cold sinter anneal at 900 °C in N_2_.

Using LLZAO as a flux/binder, LCO-LLZAO ceramics were successfully fabricated with a high relative density of >95% at a low sintering temperature of 200 °C, with a pressure of 630 MPa and water as the transient liquid. The LLZAO phase disperses homogeneously in the LCO matrix without forming any significant blocking layer(s). A highly textured surface (LF = 0.91) was observed after CSP, but the level of orientation decreased with depth into the ceramic. Electrical characterization, including impedance spectroscopy and 4-probe resistivity at various temperatures, shows enhanced electronic conductivity compared to LCO ceramics synthesized via other routes and is primarily attributed to partial oxidation of Co-ions due to Li loss during CSP of LCO-LLZAO. A one order of magnitude anisotropy between in-plane and out-of-plane conductivity was obtained. The temperature-dependent conductivity was consistent with 3D VRH and normal (Arrhenius) activation-type behavior for sub-ambient and elevated temperatures, respectively.

## Supporting Information Available

VRH and Arrhenius-type plots for conductivity measured by diffrerent experimental setups (PDF)
