# Supplementary material for: Aqueous Cold Sintering of Li-Based Compounds
Source: ACS Appl Mater Interfaces. 2023 Apr 13;15(16):20228–39. doi: 10.1021/acsami.3c00392 (PMC10141261; doi:10.1021/acsami.3c00392)
Supplement: Supplementary file 1 — am3c00392_si_001.pdf [file am3c00392_si_001.pdf]

Aqueous cold sintering of Li-based compounds

Linhao Li[a,b], Jessica Andrews[b], Ria Mitchell[b], Daniel Button[b], Derek C. Sinclair[b] and Ian M. Reaney[b*]

[a] College of Mathematics and Physics
Beijing University of Chemical Technology
Beijing, 100029, China

[b]Department of Materials Science and Engineering
University of Sheffield
Mappin St
Sheffield, S1 3JD, UK

* Corresponding author. E-mail address: i.m.reaney@sheffield.ac.uk

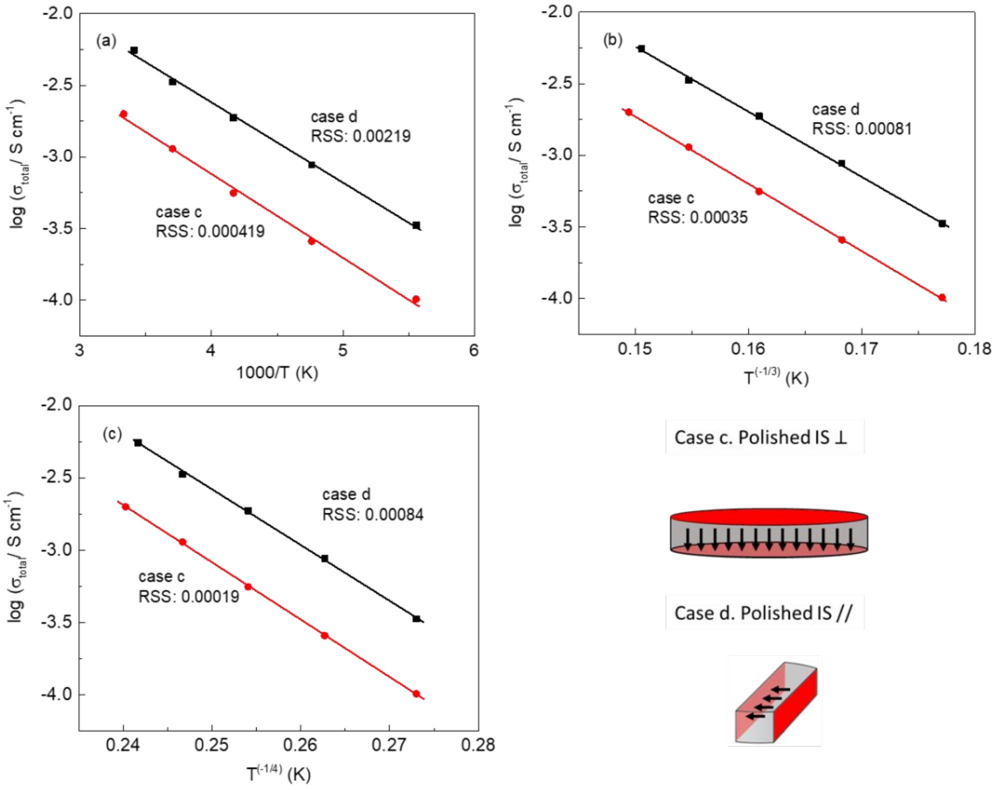

Figure S1. Impedance in/out plane total conductivity (Case c and d) plotted against a) 1000/T, b) $T^{-1/3}$ and c) $T^{-1/4}$. (RSS: Residual Sum of Squares)

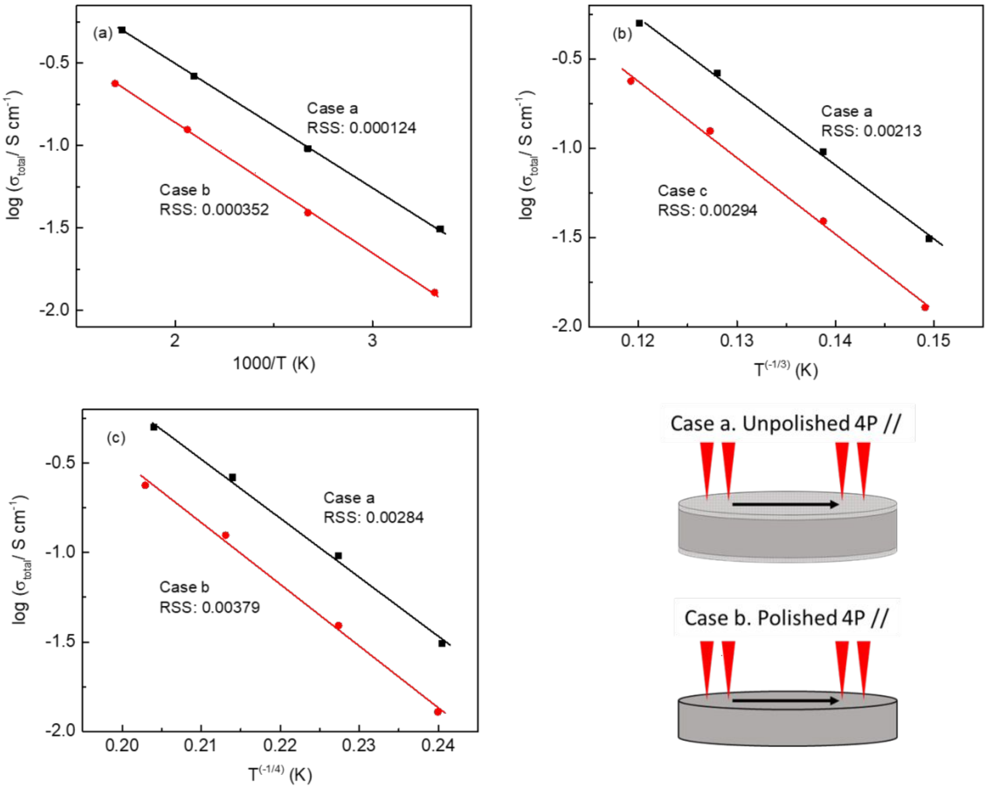

Figure S2. Four-probe, in plane total conductivity (Case a and b) plotted against a) 1000/T, b) $T^{-1/3}$ and c) $T^{-1/4}$. (RSS: Residual Sum of Squares)

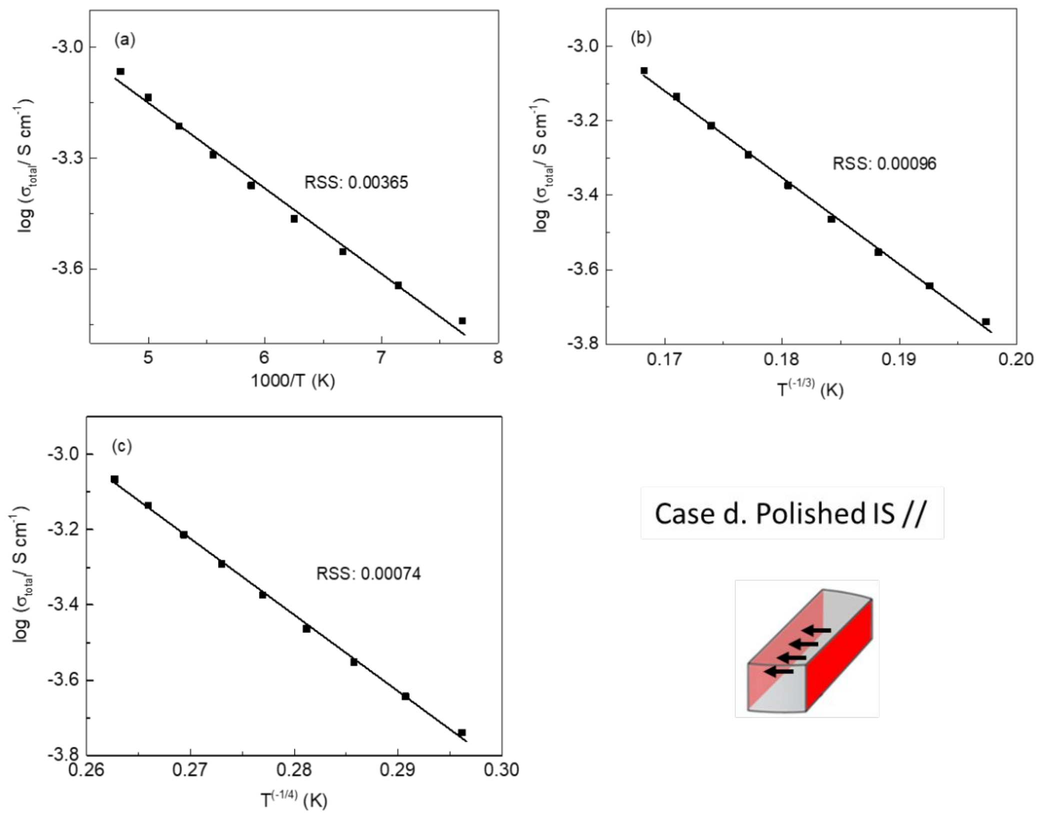

Figure S3. Impedance in plane total conductivity (Case d) plotted against a) 1000/T, b) $T^{-1/3}$ and c) $T^{-1/4}$. (RSS: Residual Sum of Squares)
